# Evaluation of diurnal and postural intracranial pressure employing telemetric monitoring in idiopathic intracranial hypertension

**DOI:** 10.1186/s12987-022-00384-2

**Published:** 2022-11-01

**Authors:** James L Mitchell, Rebecca Buckham, Hannah Lyons, Jessica K Walker, Andreas Yiangou, Matilde Sassani, Mark Thaller, Olivia Grech, Zerin Alimajstorovic, Marianne Julher, Georgios Tsermoulas, Kristian Brock, Susan P Mollan, Alexandra J Sinclair

**Affiliations:** 1 Institute of Metabolism and Systems Research, College of Medical and Dental Sciences, University of Birmingham, B15 2TT Birmingham, UK; 2 Centre for Endocrinology, Diabetes and Metabolism, Birmingham Health Partners, B15 2TH Birmingham, UK; 3 Department of Neurology, University Hospitals Birmingham NHS Foundation Trust, Queen Elizabeth Hospital, B15 2WB Birmingham, UK; 4 Department of Neurosurgery, Queen Elizabeth Hospital Birmingham, University Hospitals of Birmingham, Birmingham, UK; 5 Cancer Research Clinical Trials Unit, University of Birmingham, Birmingham, UK; 6 Birmingham Neuro-Ophthalmology, University Hospitals Birmingham NHS Foundation Trust, Queen Elizabeth Hospital, B15 2WB Birmingham, UK; 7 Department of Neurosurgery, Rigshospitalet, Copenhagen, Denmark; 8 Academic Department of Military Rehabilitation, Defence Medical Rehabilitation Centre, Stanford Hall, LE12 5BL Loughborough, UK

**Keywords:** Idiopathic intracranial hypertension, Intracranial pressure, Telemetric monitor, Diurnal, Posture

## Abstract

**Objectives:**

Intracranial pressure (ICP) has been thought to vary diurnally. This study evaluates diurnal ICP measurements and quantifies changes in ICP occurring with changes in body posture in active idiopathic intracranial hypertension (IIH).

**Methods:**

This prospective observational study utilized telemetric ICP monitoring in people with active IIH. Participants had the Raumedic p-Tel ICP intraparenchymal device (Raumedic, Hembrechts, Germany) surgically inserted. Changes in ICP in the supine position were evaluated. Then, the ICP was measured in the standing, sitting, supine, left lateral decubitus positions and with coughing and bending. Ultimately, changes in ICP over the course of 24 h were recorded. ISRCTN registration number 12678718.

**Results:**

15 women were included, mean (standard deviation) age 29.5 (9.5) years, body mass index 38.1 (6.2) kg/m^2^, and baseline mean ICP of 21.2 (4.8) mmHg (equivalent to 28.8 (6.5) cmCSF). Mean ICP rose with the duration in the supine position 1.2 (3.3) mmHg over 5-minutes (p = 0.175), 3.5 (2.8) mmHg over 30-minutes (p = 0.0002) and by a further 2.1 (2.2) mmHg over 3 h (p = 0.042). Mean ICP decreased by 51% when moving from the supine position to standing (21.2 (4.8) mmHg to 10.3 (3.7) mmHg respectively, p = 0.0001). Mean ICP increased by 13% moving from supine to the left lateral decubitus position (21.2 (4.8) mmHg to 24.0 (3.8) mmHg, p = 0.028). There was no significant difference in ICP measurements at any point during the daytime, or between 5-minute standing or supine recordings and prolonged ambulatory daytime and end of night supine recordings respectively. ICP, following an initial drop, increased progressively in conjunction with lying supine position from 23:00 h to 07:00 h by 34% (5.2 (1.9) mmHg, p = 0.026).

**Conclusion:**

This analysis demonstrated that ICP does not appear to have a diurnal variation in IIH, but varies by position and duration in the supine position. ICP rose at night whilst the patient was continuously supine. Furthermore, brief standing and supine ICP measures in the day predicted daytime prolonged ambulatory measures and end of night peak ICP respectively. This knowledge gives reassurance that ICP can be accurately measured and compared at any time of day in an ambulant IIH patient. These are useful findings to inform clinical measurements and in the interpretation of ICP analyses in IIH.

**Trial registration:**

ISTCRN (12678718).

## Background

Idiopathic Intracranial Hypertension (IIH) is characterized by raised intracranial pressure (ICP) which causes papilledema bringing the risk of permanent visual loss and chronic headaches which significantly diminish quality of life [1, 2]. IIH is becoming increasingly common, in part due to the rising obesity rates worldwide [3]. Disease modification is achieved through weight loss and is the mainstay of management [4]. However, for a small portion of patients, urgent neurosurgical intervention is warranted to save vision [1–3]. The use of intraparenchymal ICP monitoring for management of complex IIH is increasing [5–8]. These monitors allow accurate and repeated quantification of ICP, either in the routine or urgent clinical setting, which can inform management [9, 10].

Appropriate interpretation of ICP telemetric data is crucial, but not trivial. It necessitates a detailed understanding of factors that may alter ICP during the course of the recording, such as posture, specific maneuvers (e.g. coughing or bending) and time of day.

Previous work has identified that ICP does alter with posture [11, 12], but such changes may be disease-specific and affected by the time spent in a particular posture; moreover, quantification in IIH is lacking.

Patients frequently report severe worsening of their headache following coughing or bending which potentially relate to prolonged raised ICP caused by a cough or bend. This phenomenon, although clinically relevant, has not been previously characterized in IIH.

Some have suggested that ICP may vary with the circadian rhythm, for example higher ICP was reported in a patient with hydrocephalus at night [8]. However, it is currently unknown whether there are ICP diurnal and nocturnal changes in IIH.

All these factors need to be considered when interpreting telemetric results and understanding of how they contribute to ICP is relevant to designing research and clinical protocols. At present, there is no consensus on optimal length of recording which would constitute an appropriate and unbiased representation of overall ICP over time. Identification of optimal length of recording is a necessary step towards standardization in clinical practice and research.

This prospective observational study aimed to characterize ICP dynamics in IIH and evaluate whether shorter-lasting ICP measurements conducted in the supine position are a reliable predictor of ICP over the night and whether brief standing position measurements predict daytime prolonged ambulatory ICP measures.

To provide a comprehensive picture of ICP changes in IIH, we measured ICP variations with changing posture (standing, sitting, supine, and lateral decubitus position) and included the relationship between ICP and amplitude as an indirect indicator of compliance. We assessed whether the time spent in the supine position had an impact on mean ICP, characterized ICP kinetics with coughing and bending and quantified changes in mean ICP over the course of 24 h (to investigate diurnal and nocturnal variations). Those last recording were also used to test the hypothesis that shorter measurements of ICP acquired in the standing and supine positions are representative of mean daytime and nighttime ICP, respectively.

## Methods

This prospective observational study was approved by the West Midlands Solihull Research Ethics Committee (17/WM/0179). Participants were recruited via the IIH clinical service, University Hospitals Birmingham NHS Foundation Trust and the trial was registered with ISTCRN (12678718). In accordance with the Declaration of Helsinki, all subjects gave written informed consent to participate in the study.

All women between 18 and 60 years were eligible if they had a clinical diagnosis of active IIH meeting the IIH consensus guidance [2] which included ICP ≥ 25 cmCSF as measured by lumbar puncture (LP) in the lateral decubitus position; papilledema as diagnosed by a neuro-ophthalmologist; and normal brain imaging, except for findings suggestive of raised ICP. Papilledema was graded by a neuro-ophthalmologist using the Frisén classification [13], as part of the eligibility criteria for study inclusion.

This included the explicit exclusion of venous sinus thrombosis with either magnetic resonance venography or CT venography. Body Mass Index (BMI) was measured from height and weight using the formula: BMI = weight(kg) / (height (m))^2^. Participants on ICP modifying medicines, such as acetazolamide or topiramate, discontinued these for one month prior to enrolment.

Intracranial pressure is defined as the static pressure within the skull relative to atmospheric pressure, in this study the Raumedic Transdermal Telemetric System (Raumedic, Hembrects, Germany) was used for the measurement of ICP. The Neurovent P-tel, which is a fully implantable telemetric sensor, was inserted surgically in the right frontal area under general anaesthesia as a day-case procedure. The ICP was recorded with the reader (Reader TDT1 readP) placed over the implanted catheter and saved to the portable storage unit of the system (Datalogger MPR1). Both positive and negative readings were acquired. The sampling frequency of the system is 5 Hz, this frequency was used for all recordings. Recordings were made in mmHg and converted into cmCSF using a 1.36 conversion factor.

ICP measures were recorded over a two-day research visit, during which the participants were able to move freely within the medical facility. ICP was recorded continuously over a 5-minute, 30-minute and 3-hour period with participants adopting a supine posture. ICP measurements were then acquired with the participants in a supine, lateral decubitus, sitting and standing position for 5-minutes each. The supine position was 0° to horizontal without additional head support, the lateral decubitus position with hips flexed to 90° and knees flexed; the neck was in a neutral position with the head supported to maintain neutral neck alignment. Sitting measurements were acquired with the subject unsupported on the edge of a hospital bed with legs dependent, and standing measures were recorded upright in a relaxed pose. This was followed by the participant performing two bends and two coughs. For the bending measurements, participants were instructed to flex from the hips whilst standing and attempt to touch the floor before returning to standing. In order to evaluate coughing, subjects were instructed to perform a single maximal effort cough. Following each individual bend and cough, participants paused for 1-minute. Bend and cough waveforms were marked manually. Prolonged ambulatory ICP was recorded by allowing participants to take the equipment home, participants were instructed to record continuously for as long as possible, whilst undertaking normal activity.

To assess diurnal and nocturnal ICP, recordings were made at pre-specified timepoints whilst the participant was awake (11:00 h, 13:30 h, 17:00 h and 23:00 h ± 1 h). Each recording was made in the supine position for 30-minutes. Between recordings, participants were ambulant and participated in activities of daily living. ICP was then recorded continuously overnight (23:00 to 07:00 h) whilst the patient slept (natural lying sleeping postures being permitted).

### Intracranial pressure analysis

The recordings were analyzed with the software Dataview version 1.2 (Raumedic, Hembrects, Germany). The full duration of the recording was analyzed, and the mean ICP over the specified time-period was calculated. Amplitude was defined as the difference between the peak and the trough of each ICP cardiac wave. The proprietary software evaluated the amplitude for each peak over a 5-minute ICP recording interval and provided a mean value, which we have referred to as amplitude in this study. Hence the amplitude of multiple peaks was measured, and the mean calculated during the measurement period. Amplitude values were subdivided into three groups on the basis of mean ICP measurements: mean ICP < 18.4 mmHg; between 18.4 and 22.1 mmHg and > 22.1 mmHg; corresponding to < 25 cmCSF, 25–30 cmCSF and > 30 cmCSF, respectively. Groups were then compared to evaluate intracranial pressure and ICP waveform amplitude. We did not take into account ICP drift of the system as this has been previously evidenced as minimal − 3 mmHg over 2 years.[14] We monitored for ICP shifts that would indicate unreliable results.

Statistical evaluation was performed with R 4.0.0 (R foundation for statistical computing) and GraphPad Prism 8.0 (GraphPad Software), outcomes were summarized by means with standard deviation (SD) or median and IQR for non-parametric data, normality was assessed using the Shapiro-Wilk test. Values were compared using t-tests, ANOVA or hierarchical regression as indicated. *Hierarchical linear regression models were used to analyse repeated measures of ICP and to estimate differences adjusted for baseline values. In these models, population-level effects (also known as fixed effects) comprised the intercept, time as a factor variable, and the interaction of cohort and time to model group-specific means effects through time. Adjusting for baseline values was achieved through the use of subject-specific intercepts.* Missing data was excluded. The level of significance was set at P < 0.05. This was a prospective evaluation, and we report the primary analysis of the data.

## Results

The mean (SD) age of the 15 women was 28 (9.0) years, BMI was 38.1 (6.2) Kg/m^2^, mean ICP measured supine was 23.5 (3.9) mmHg which corresponds to 32.0 (5.3) cmCSF. Frisén grade was median (interquartile range) 2 (1) (Table 1). The median (IQR) time from surgery to baseline visit was 10 (16.5) days. There were no surgical complications with the exception of minor superficial site infection. ICP recordings displayed no measurement shifts indicative of recording error over the duration of the study and hence no recordings needed exclusion.

**Table 1:** Baseline characteristics

	Mean (SD), n = 15
**Age (years) (all female)**	28 (9)
**BMI (kg/m** ^**2**^ **)**	38.1 (6.2)
**ICP as measured with Raumedic (mmHg) supine**	21.2 (4.8)
**ICP converted to cmCSF (cmCSF)**	28.8 (6.5)
	**Median (IQR)**
Frisén grade*	2 (2–3)

BMI indicates body mass index, ICP indicates intracranial pressure, SD indicates standard deviation. * The star indicates grading the worst eye

### Intracranial pressure variations with changing position

**Fig. 1:**
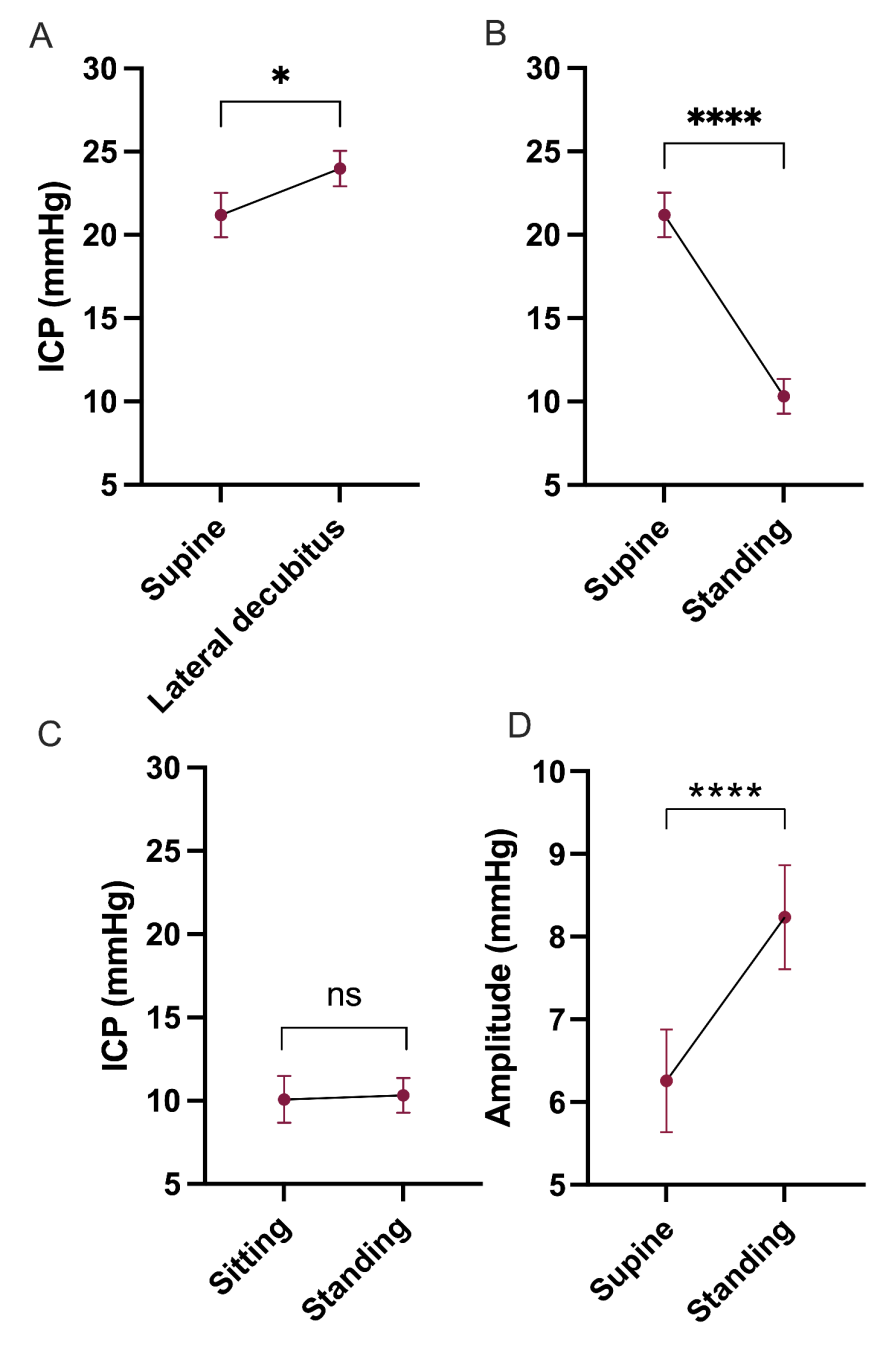
Intracranial pressure changes occurring as a result of altering posture ICP was recorded in the supine, lateral decubitus, sitting and standing positions. Graphs show ICP in mmHg against (A) supine and lateral decubitus, (B) supine and standing, (C) sitting and standing, (D) Amplitude increases from supine to standing (p < 0.0001). n = 15 (A-D). Paired t-test. Data presented as mean ± standard error of the mean. ns = non-significant, *P = < 0.05, ****P = < 0.0001

The effect of position on ICP was evaluated. In the supine position mean (SD) ICP was 21.2 (4.8) mmHg, in the lateral decubitus position 24.0 (3.8) mmHg, sitting 10.1 (5.1) mmHg and standing 10.3 (3.7) mmHg (Table 2). Comparing the changes from supine to lateral decubitus position mean ICP rose 2.8 (4.0) mmHg, there was a 13% change, (p = 0.028); from supine to standing mean ICP fell by 10.9 (4.2) mmHg, equivalent to a -51% change, (p = 0.0001). Amplitude rose significantly between supine and standing, p = < 0.0001 all values. There was no significant change between sitting and standing, 0.2 (3.8) mmHg, 2% change, (p = 0.82). (Tables 2 and 3; Fig. 1)

**Table 2:** Changes in intracranial pressure resulting from altering position

Position change	ICP change mean (SD) (mmHg)	Mean percentage change (%)	p= (paired t-test)
**Supine to LP**	2.8 (4.0)	13%	0.028*
**Supine to standing**	-10.9 (4.2)	-51%	0.0001****
**Sitting to standing**	0.2 (3.8)	2%	0.82
**Standing vs. ambulatory recording**	-1.3 (1.1)	3%	0.39

ICP indicates intracranial pressure, SD indicates standard deviation, LP indicates lateral decubitus position, * indicates p = < 0.05, **** indicates p = < 0.0001

**Table 3:** Change in intracranial pressure waveform resulting from altering posture

	Supine mean (SD)	Standing mean (SD)	Mean percentage change (%)	p= (paired t- test)
**Peak pressure (mmHg)**	27.5 (6.7)	16.4 (4.0)	-40.4	< 0.0001 ****
**Trough pressure (mmHg)**	20.1 (4.4)	8.2 (3.0)	-59.2	< 0.0001 ****
**Amplitude (mmHg)**	6.3 (2.1)	8.2 (2.2)	30.2	< 0.0001****
**Cardiac rate (bpm)**	69.4 (10.7)	80.2 (11.0)	15.6	< 0.0001****

SD indicates standard deviation, bpm indicates beats per minute, **** indicates p = < 0.0001

ICP recorded during prolonged daytime ambulatory (standing) monitoring was compared to that recorded in the standing position over 5-minutes, the standing ambulatory ICP mean (SD) was 10.2 (3.4) mmHg and was not significantly different to standing mean (SD) ICP measured over 5-minutes 10.3 (3.74) mmHg with 3% variation, p = 0.39, between the mean values. (Table 2) In addition for an individual patient we observed little variability between the prolonged daytime ambulatory (standing) monitoring and that recorded in the standing position over 5-minutes. (Table 4)

**Table 4:** Standing versus ambulatory intracranial pressure individual comparisom

Participant	Ambulatory mean ICP (mmHg)	Standing mean ICP (mmHg)	Difference (mmHg)
**1**	6.62	5.2	-1.42
**4**	6.96	5.78	-1.18
**5**	9.79	11.75	1.96
**7**	12.55	12.9	0.35
**9**	10.61	10.5	-0.11
**10**	8.62	9.25	0.63
**11**	7.39	6.99	-0.4
**13**	15.07	13.36	-1.71
**14**	8.14	6.91	-1.23
**16**	16.44	16.34	-0.1

ICP indicates intracranial pressure

**Fig. 2:**
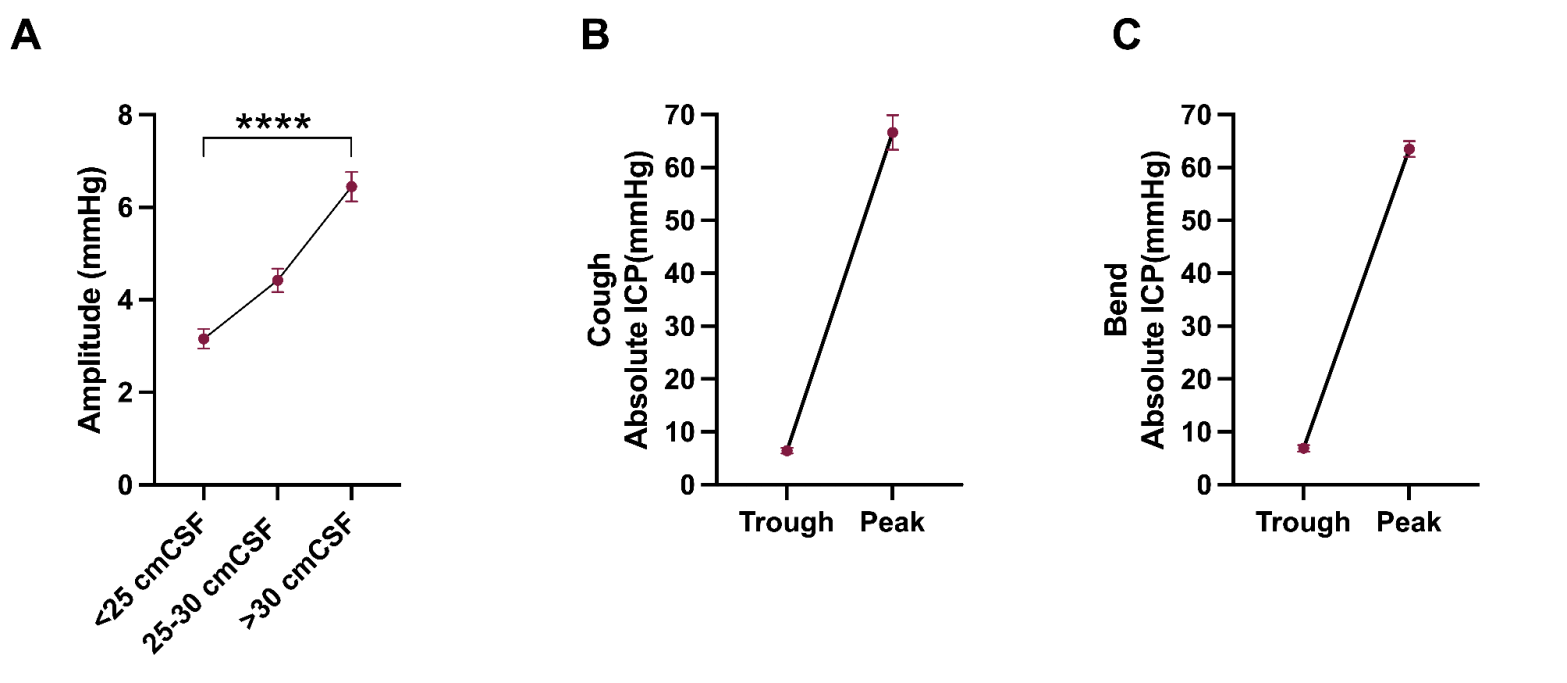
Intracranial pressure waveform changes characterized by different categories of ICP and occurring due to coughing and bending A) Patients ICP recordings were grouped by mean ICP into < 18.4 mmHg (25 cmCSF), 18.4–22.1 mmHg (25–30 cmCSF) and > 22.1 mmHg (30 cmCSF). As mean ICP increases amplitude of the waveform increases significantly (p < 0.0001). ANOVA. Data presented as mean ± standard error of the mean. *P = < 0.05, ****P = < 0.0001. ICP was recorded during the performance of coughing and bending. Graphs show trough to peak changes in ICP in mmHg with cough (B) and bend (C), showing absolute ICP changes in ICP. n = 15 (B-C. Paired t-test. Data presented as mean ± standard error of the mean

Between each ICP category (< 18.4, between 18.4 and 22.1 and > 22.1 mmHg) the amplitude increased with overall mean ICP (p < 0.0001, Table 5; Fig. 2) suggesting that those patients with higher ICP also had increased amplitude and potentially reduced compliance.

**Table 5:** Waveform changes with mean ICP

	Normal (< 18.4 mmHg) mean (SD)	Elevated (18.4–22.1 mmHg) mean (SD)	High (> 22.1 mmHg) mean (SD)	p= (ANOVA)
**Peak pressure (mmHg)**	16.23 (2.7)	22.4 (1.2)	30.7 (3.2)	< 0.0001 ****
**Trough pressure (mmHg)**	13.1 (2.2)	18.0 (0.7)	24.2 (2.7)	< 0.0001 ****
**Amplitude (mmHg)**	3.2 (0.8)	4.4 (1.2)	6.4 (1.7)	< 0.0001****
**Cardiac rate (bpm)**	60.9 (10.0)	64.0 (9.5)	70.6 (12.6)	0.0229*

SD indicates standard deviation, ANOVA indicates analysis of variance, bpm indicates beats per minute, * indicates p = < 0.05, **** indicates p = < 0.0001

### Effect of coughing and bending on intracranial pressure

Cough had a marked effect on ICP. The mean (SD) peak pressure during cough increased from 14.9 (5.8) mmHg to 66.7 (27.2) mmHg, 343.0% change, (p < 0.0001, Table 6). The amplitude increased from 7.5 (2.4) mmHg to 60.2 (25.8) mmHg, 696.0% change, (p < 0.0001, Table 6). This was equivalent to a tenfold change from the point of inflection in the waveform to the peak during the cough. The duration for ICP to return to baseline following a cough was 3.7 (1.5) seconds. Evaluation of the ICP trace following a cough noted that there was a negligible change in mean ICP when comparing the pre to the post baseline ICP, with a change of -0.7 (4.5) mmHg (peak pressure − 1.0 (4.6) mmHg and trough pressure − 0.5 (3.8) mmHg). The amplitude was reduced comparing the pre to the post baseline ICP by -0.5 (1.7) mmHg, a 6.6%, reduction (p = 0.0009, Table 6, ,Fig. 2).

**Table 6:** Intracranial pressure changes with cough

	Before Mean (SD)	During Mean (SD)	After Mean (SD)	Change (before to during) Mean (SD)	% Change (before to during)	P value (before-during)	Change* (before to after) Mean (SD)	% Change* (before to after)	p Value* (before to after)
**Mean (mmHg)**	11.6 (5.0)	-	10.9 (5.4)	-	-	-	-0.7 (4.5)	-5.9	0.2201
**Peak (mmHg)**	14.9 (5.8)	66.7 (27.2)	13.8 (6.0)	51.1 (26.7)	343.0	< 0.0001	-1.0 (4.6)	-7.0	0.0704
**Trough (mmHg)**	7.4 (4.3)	-	6.8 (4.6)	-	-	-	-0.5 (3.8)	-7.2	0.2511
**Amplitude (mmHg)**	7.5 (2.4)	60.2 (25.8)	7.0 (2.2)	52.2 (25.3)	696.0	< 0.0001	-0.5 (1.7)	-6.6	0.0009**

The table shows results of waveform analysis of patient performing a single maximal cough, columns 2–4 show mean ICP before the inflexion of the ICP rise due to the bending event, during the event and following the inflexion at the end of the event. Columns 5–7 show comparison between the values before and during the event. Columns 8–10 show comparison between values before and after the event

During bending, mean (SD) peak pressure increased by 48.8 (13.3) mmHg, from 15.1 (6.1) mmHg to 63.5 (12.4) mmHg, a 323.2% change, (p < 0.0001,Table 7). The amplitude of the waveform increased by 49.3 (13.2) mmHg, from 7.7 (2.4) mmHg to 56.6 (12.7) mmHg, a 640.3% change, (p < 0.0001,Table 7). There was a 9.2-fold change in ICP from the point of inflection to the peak. It took 4.1 (2.4) seconds for ICP to return to baseline following a bend, with none of the waveform parameters changing significantly when comparing the pre to the post ICP trace (Fig. 2).

**Table 7:** Intracranial pressure changes with bending

	Before Mean (SD)	During Mean (SD)	After Mean (SD)	Change Mean (SD) (before to during)	% Change (before to during)	P Value (before to during)	Change Mean (SD) (before to after)	% Change (before to after)	P Value (before to after)
**Mean (mmHg)**	11.9 (5.4)	-	12.4 (5.1)	-	-	-	0.5 (4.3)	4.2	0.3431
**Peak (mmHg)**	15.1 (6.1)	63.5 (12.4)	15.7 (5.7)	48.8 (13.3)	323.2	< 0.0001	0.6 (4.7)	4.2	0.2763
**Trough (mmHg)**	7.5 (4.6)	-	7.9 (4.6)	-	-	-	0.5 (3.6)	6.4	0.2808
**Amplitude (mmHg)**	7.7 (2.4)	56.6 (12.7)	7.8 (2.3)	49.3 (13.2)	640.3	< 0.0001	0.2 (1.8)	2.0	0.3182

The table shows results of waveform analysis of patient bending at the waist to touch ground and returning to standing posture, columns 2–4 show mean ICP before the inflexion of the ICP rise due to the bending event, during the event and following the inflexion at the end of the event. Columns 5–7 show comparison between the values before and during the event. Columns 8–10 show comparison between values before and after the event

### Diurnal and nocturnal intracranial pressure variations

**Fig. 3:**
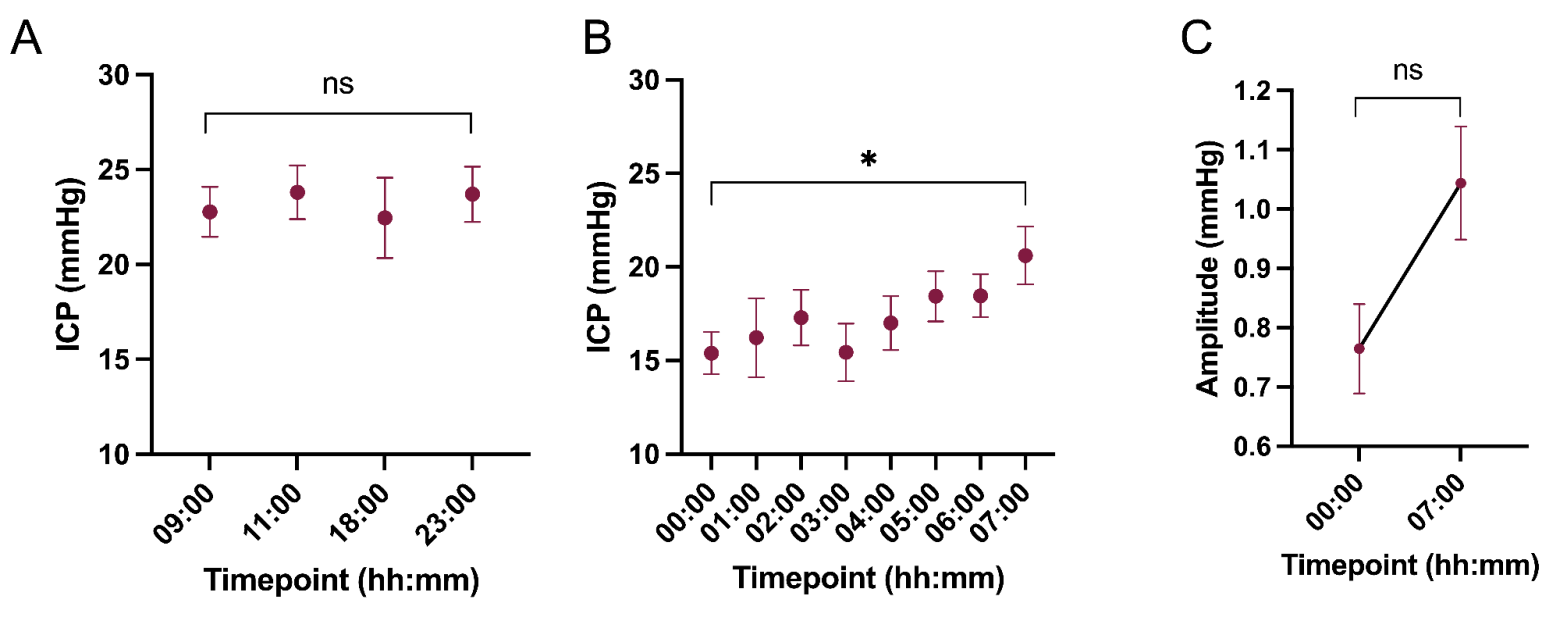
Changes over 24 h (A) ICP was recorded supine at set timepoints throughout waking hours. Graph shows ICP in mmHg at 09:00, 11:00, 18:00 and 23:00. (B) ICP in mmHg was recorded overnight whilst supine, between 23:00 and 07:00, each value represents recorded mean ICP over the preceding hour. n = 6. Hierarchical regression. (C) Amplitude in mmHg at the beginning of the night recording and at the end of the night. Paired t test. Data presented as mean ± standard error of the mean. ns = non-significant, *P = < 0.05

ICP was evaluated over a 24-hour period in 7 participants. There was no significant change in mean ICP between 09:00 and 23:00 (mean daytime supine ICP 23.6 mmHg, (SD 4.0)). The patient then retired for the night. Following a drop in mean ICP, observed at the beginning of the night recording, ICP steadily rose overnight whilst the patient slept in their usual sleeping position, equivalent to a 34% rise (between 00:00 h, mean (SD) 15.4 (2.5) mmHg, and 07:00 h 20.6 (3.4) mmHg, p = 0.026) (Table 8; Fig. 3). The mean daytime supine mean ICP and end of night supine mean ICP (at 0700 h) were not significantly different (p = 0.102). There was a trend toward higher amplitude (p = 0.06) at the end of the night possibly indicating that compliance changed in tandem with ICP overnight (Fig. 3).

**Table 8:** Changes in ICP over 24 h

Timepoint (hh:mm) n = 7	ICP mean (SD) (mmHg)	Difference baseline to timepoint mean (SD); (95%CI)	p (Hierarchical regression)
All day timepoints	23.6 (4.0)	**-**	**-**
09:00	22.8 (3.5)	**-**	
11:00	23.8 (3.7)	1.0 (2.2); (-1.0, 3.0)	p = 0.259
18:00	22.5 (5.6)	-0.3 (3.2); (-3.3, 2.7)	p = 0.801
23:00	23.7 (3.9)	0.9 (1.5); (-0.5, 2.3)	p = 0.155
All night timepoints	17.3 (3.7)	-	-
00:00 (midnight)	15.4 (2.5)	-	
01:00	16.2 (5.2)	0.8 (2.5); (-4.9, 6.6)	p = 0.754
02:00	17.3 (3.6)	1.9 (1.9); (-2.5, 6.3)	p = 0.354
03:00	15.4 (3.8)	0.0 (2.0); (-4.5, 4.5)	p = 0.986
04:00	17.0 (3.5)	1.6 (1.9); (-2.7, 5.9)	p = 0.417
05:00	18.4 (3.3)	3.0 (1.8); (-1.0, 7.1)	p = 0.125
06:00	18.5 (2.8)	3.1 (1,6); (-0.6, 6.7)	p = 0.090
07:00	20.6 (3.4)	5.2 (1.9); (0.8, 9.6)	p = 0.026*

ICP indicates intracranial pressure, SD indicates standard deviation, CI indicates confidence, intervals, * indicates p = < 0.05

### Effect of length of measurement on intracranial pressure results

**Fig. 4:**
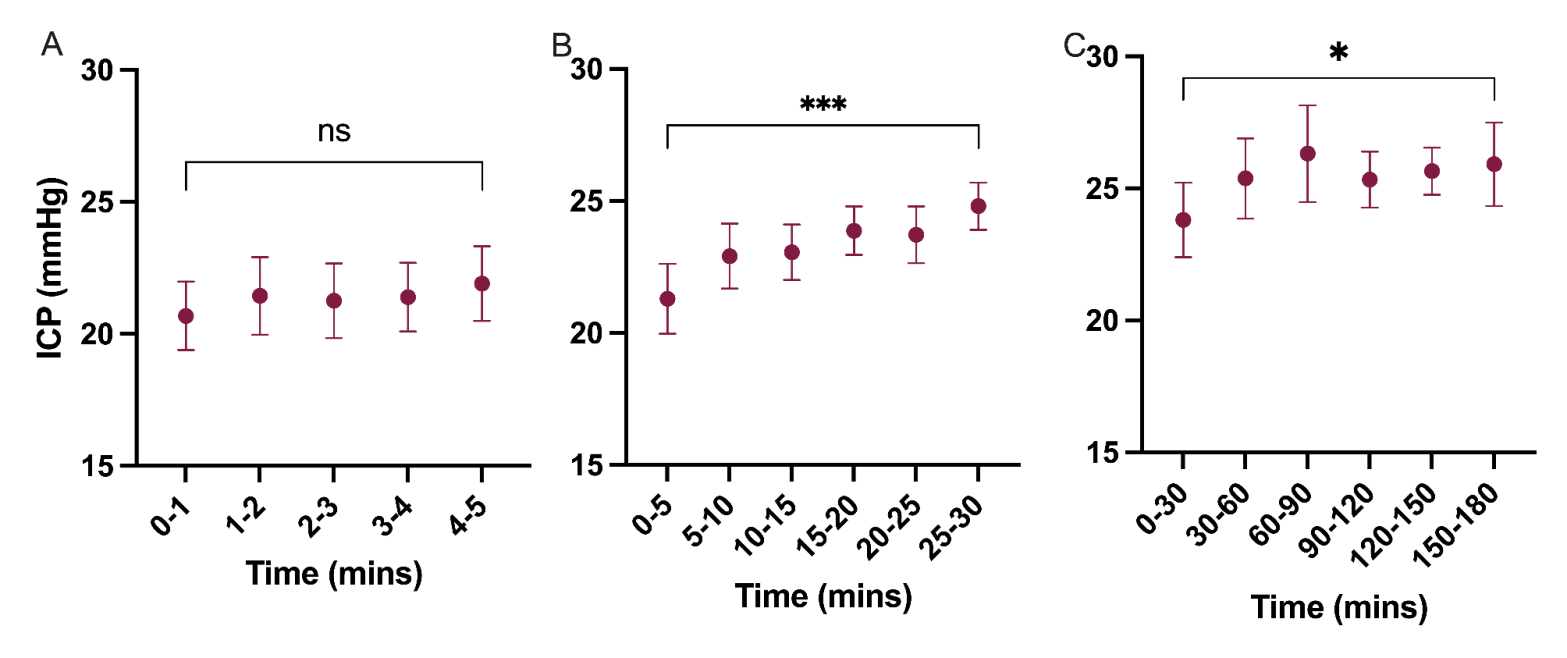
Intracranial pressure changes occurring over the duration of recording ICP was recorded over differential periods in the supine position A) over 5-minutes (p = non-significant), B) over 30-minutes (p = 0.0002), C) over 3 h (0 = 0.042), values expressed in mmHg. n = 15 (A-B) and n = 7 (C). Paired t-test. Data presented as mean ± standard deviation. ns = non-significant, *P = < 0.05, ***P = < 0.001

In the supine position, mean ICP did not significantly change over 5-minutes (Fig. 4). Over 30-minutes of recording, mean ICP rose significantly (mean (SD)), by 3.5 (2.8) mmHg; p = 0.0002) equivalent to a 16% increase; (Fig. 4). From 30-minutes to 3 h the mean ICP increased by an additional 2.1 (2.2) mmHg (p = 0.042), with the total rise in mean ICP being equivalent to 25%; (Fig. 4).

## Discussion

We present an in-depth characterization of postural, diurnal and nocturnal ICP variations in active IIH, including assessments of the impact of length of recording on results and changes in amplitude, a reflection of intracranial compliance.

Diurnal ICP in IIH was markedly elevated compared to expected values in a pseudo-normal population[15] and we provided evidence that brain compliance decreased with rising ICP; these findings are consistent with current literature[8, 16] and offer reassurance on the robustness of these telemetric measurements. We observed that ICP varied according to position and duration in that position. Importantly, we have quantified the differences in ICP occurring as a patient moved between the positions of standing, sitting, supine and the lateral decubitus (Fig. 1). We have confirmed previous findings that ICP rises when adopting the lateral decubitus position and that ICP falls in both the sitting and standing positions [11, 12, 16, 17]. We have further quantified these changes and report clinically useful summary data. Mean ICP reduced by 51% moving from supine to standing and increased by 13% when changing from supine to the lateral decubitus position in IIH. It is also important to note that there was no significant difference between the sitting and standing positions, and hence recording ICP in both these positions may be unnecessary. Additionally, this is useful to note when considering that the clinical examination of papilledema, such as by optical coherence tomography is performed in the sitting position [18].

*Andresen et al.* previously observed that ICP measurement in the lateral decubitus position was higher than in supine position, a finding we have confirmed [19]. Detailed work by *Pedersen et al.* has shown that the measurement of ICP in the left lateral decubitus position is affected by small changes in posture of the neck and hips having marked effects on the recorded ICP [20]. Most recently *Norager et al.* have reported a systematic review aiming to establish normative cut-offs for both lumbar cerebrospinal fluid (CSF) pressure and supine ICP [21]. Although the IIH diagnostic criteria recommend that ICP should be measured with hips flexed to 90° [22], there is frequent difficulty in performing LPs in IIH due to abdominal adiposity limiting hip flexion [23–25]. It is an important clinical message that variability in the lateral decubitus position can lead to clinically relevant changes in ICP. Therefore, medical professionals should be cognizant of the position the patient adopts and, where possible, pay particular attention to this standardized position.

We have characterized changes in ICP upon coughing and bending, two activities anecdotally reported to significantly worsen headache in IIH. ICP increased substantially during both activities, but, reassuringly, returned to baseline levels rapidly, demonstrating that coughing or bending do not cause prolonged elevation in ICP. We also noted a significant reduction in amplitude in the ICP waveform following a cough.

Mean diurnal ICP was relatively stable, whereas there were significant alterations in the night recordings. Mean ICP declined sharply at the beginning of the night measurements (at the point of sleep onset) and then rose slowly whilst the patient was lying asleep at night. The last nocturnal recording prior to waking up being equivalent to (but not higher than) mean daily ICP whilst supine. The observed rise in mean ICP overnight is of interest, headache due to raised ICP is typically noted to be worse on waking whilst the patient is supine and to regress as the patient becomes ambulant in the day (with ICP falling in the standing position). Rising ICP overnight has not be observed in other conditions and may be a specific finding in IIH [8, 16, 26–31].

We have found no significant daytime variability in mean ICP in this cohort of patients with active IIH, whilst the patients are in a comparable position (supine). Such an increase over night has not been previously reported in IIH and might be a pathological characteristic of IIH. There was a trend toward raised amplitude at the end of the night, possibly indicating that compliance decreased with rising ICP overnight.

There are previous studies of ICP during sleep. [8, 16, 26–31] The only study reporting analysis of ICP over the duration of sleep was *Ogashiwa et al.* in a single case of a patient with Chiari malformation where pressure was observed to rise until a newly inserted shunt valve was opened and thereby dropping pressure [26].

We have demonstrated rising mean ICP over the course of a prolonged period of lying supine at night. We also observed that the ICP amplitude was lower whilst asleep during the overnight recording compared to that observed whilst awake during the day. The underlying mechanism has not been explored in this study but would be of future interest. For instance, a consequent question would be whether such night fluctuations in ICP are related to the circadian rhythm, (analogously to cortisol, blood pressure and cerebral perfusion [32]). Alternatively, the nocturnal changes could reflect changes in cerebral vasodilation associated with patients falling asleep; or, alternatively, may relate to alterations in posture (i.e. prolonged lying) altering venous return. Such hypotheses are not mutually exclusive and will need to be the subject of future investigations. It would also be important to investigate further the clinical relevance of increasing overnight ICP in IIH. Early morning headache is a classically taught feature of raised ICP [33, 34] and may be indicative of the climbing ICP following a prolonged period of being supine at night. Anecdotally, many IIH patients report sleeping sat up to mitigate early morning headache, and early morning transient visual obscurations. This observation of night-time rising ICP has the potential to permit targeted treatment to preferentially reduce nocturnal ICP.

Consensus for the duration of recording ICP is not yet established.[29] We sought to define how ICP changed according to different durations of monitoring. *Andresen et al.* utilized median pressure over a 10-minute interval following each posture change after an unspecified time period to allow the ICP to stabilize [19]. We have shown that in an IIH cohort, mean ICP rose with increasing time in the supine position. This effect was observed 30-minute and 3-hour time measurements, and is unlikely to be driven by device measurement drift.[14, 35] These observations have implications for measuring ICP over a standardized time-period, ideally 5-minutes obviating the subsequent physiologic rise in IIH. Our results support standardizing the position and duration of ICP recording for both research protocols and in clinical practice.

In the clinical environment, challenging cases may be evaluated with prolonged ICP monitoring over 24 h or even several days. Our data suggests that for an individual the mean ICP recorded over a 5-minute period whilst standing is not significantly different to the mean ICP recorded over a prolonged ambulatory period during the day. Furthermore, although ICP rises overnight whilst supine, by the end of the night the ICP supine is no higher than the ICP observed during a 5-minute recording during the day whilst supine. This data suggests that when evaluating the mean ICP a short 5-minute readings is similar to prolonged daytime ICP monitoring. However prolonged recording may reveal other ICP characteristics of clinical relevance.

The detailed analysis of the ICP amplitude revealed that changes in mean ICP were accompanied by reciprocal changes in amplitude. A key observations was that as ICP increased overnight, we also observed that amplitude rose overnight (Fig. 3). Further we observed that with higher ICP, higher amplitude was noted (Table 5). This could suggest that those with the most elevated ICP have the most compromised intracranial compliance. This would be of interest to evaluate further.

The results of this study may also help the interpretation of ICP recordings in clinical practice as there is an increasing use of telemetric ICP sensors, both as standalone devices and integrated to shunt systems. CSF diversion in patients with IIH and vision-threatening papilledema is challenging and telemetric ICP sensor is a promising tool to aid assessment and optimization of shunt function. However, the interpretation of telemetric recordings and use in clinical decision making is still limited [36]. It is possible that shunted patients have different CSF hydrodynamics compared to IIH and extrapolation of our results to other diseases and normal physiology cannot be assumed, nonetheless,the results of this study illustrate the importance of including a comprehensive assessment of ICP which is specific to the disease population prior to providing guidance for interpretation of telemetric ICP monitoring.

There are limitations to this study. Normative ICP data for direct comparison are currently lacking. This has been a challenge for all previous studies and is likely to persist owing to the ethical objection to invasive recording in healthy volunteers. Previous studies have effectively looked at pseudo-normal populations.[15] There were challenges in recording that resulted in missing data, for example due to an interruption of recording. However, as over 9000 data points were recorded at each 30-minute time-point, we are reassured that the data is representative. The sampling rate of the Raumedic system is 5Hz, in sampling ICP cardiac waves this results in a risk of underestimate and as such results should not be compared to systems with higher sampling rates, however the comparisons within this data and with the same system are valid. It is also worth considering how ICP drift could have influenced our results, however the ICP drift has been established with Raumedic p-Tel monitors as minimal, -3 mmHg over 2 years[14] and hence is unlikely to be the dominant contributor to our results.

This study identified novel dynamical ICP changes over night, but it was not designed to investigate the causes of such changes. In the future it would be important to correlate the overnight recordings with electroencephalography to assess impact of the sleep-wake cycle.

A further limitation of this study is the relatively small sample size, in particular over longer recording periods. This was due to challenges related to recruiting participants for an invasive study. However, the study does not appear to be underpowered, as demonstrated by the statistical significance and tight standard deviations. Finally, these results are only applicable to a population with active IIH.

## Conclusion

Telemetric intracranial pressure monitoring in IIH has revealed that ICP does not vary during the day, but does rise overnight following an initial drop at the time of sleep onset. We also noted marked changes in mean ICP due to changes in posture, and duration of time in the supine posture. Furthermore, brief standing and supine ICP measures in the day predicted prolonged measures of ambulatory daytime mean ICP and end of night maximal mean ICP respectively (mean of last hour of sleep ICP). These findings have relevance of how ICP should be evaluated in both a research and clinical setting.

## Data Availability

Anonymized individual participant data will be made available along with the trial protocol and statistical analysis plan. Proposals should be made to the corresponding author and will be reviewed by the Data Sharing Committee in discussion with the Chief Investigator. A formal Data Sharing Agreement may be required between respective organizations once release of the data is approved and before data can be released.

## Abbreviations

BPM: Beats per minute
CSF: Cerebrospinal fluid
CNS: Central nervous system
mmHg: Millimeter of mercury
ICP: Intracranial pressure
IIH: Idiopathic Intracranial Hypertension
LP: Lumbar puncture
OP: Opening pressure
SD: Standard deviation
